# AAV-mediated gene therapy for Alzheimer’s disease: neuroprotective mechanisms and translational challenges

**DOI:** 10.3389/fnagi.2026.1901875

**Published:** 2026-09-15

**Authors:** Zehua Yu, Yang Li, Zeyu Zhang, Rong Yin

**Affiliations:** 1 Department of Neurology, Gansu Provincial Central Hospital, Lanzhou, China; 2 School of Traditional Chinese and Western Medicine, Gansu University of Chinese Medicine, Lanzhou, China

**Keywords:** adeno-associated virus, aging, Alzheimer’s disease, amyloid-β, gene therapy, neuroinflammation, tau pathology, translational neuroscience

## Abstract

Alzheimer’s disease remains a major neurodegenerative disorder for which effective disease-modifying treatments are limited. Adeno-associated virus-mediated gene therapy provides a strategy for sustained modulation of disease-related pathways in the central nervous system. Recent studies have explored this approach for regulating amyloid-β (Aβ) metabolism, tau pathology, neuroinflammation, genetic risk factors and neurotrophic support. This review summarizes the biological basis for adeno-associated virus delivery in the central nervous system and discusses major therapeutic strategies for Alzheimer’s disease, including approaches targeting Aβ production and clearance, tau expression and degradation, immune microenvironment remodeling, apolipoprotein E modulation and synaptic protection. We further examine key translational barriers, including blood-brain barrier restriction, cross-species differences, immune responses, dose-related toxicity, manufacturing constraints and limited clinical evidence. Finally, we propose that future development should move beyond delivery efficiency alone toward biologically informed vector design, target selection, disease-stage matching and biomarker-guided clinical evaluation.

## 1 Introduction

Alzheimer’s disease (AD) is the most common cause of dementia and is biologically characterized by cerebral amyloid-β (Aβ) plaques and tau neurofibrillary pathology. These defining lesions, however, develop within a heterogeneous disease process in which synaptic dysfunction, impaired proteostasis, neuroimmune activation, and vascular and other age-related co-pathologies influence clinical presentation and progression (Knopman et al., 2021; Heneka et al., 2025). Aβ accumulation is an early and central feature of many forms of AD, particularly autosomal-dominant disease, but it does not alone explain the biological and clinical heterogeneity of sporadic late-onset AD.

Dementia affected an estimated 57.4 million people worldwide in 2019 and is projected to affect 152.8 million by 2050 (Nichols et al., 2022). Phase III trials of lecanemab and donanemab have shown that substantial amyloid lowering can modestly slow cognitive and functional decline in selected patients with early, biomarker-confirmed AD (Sims et al., 2023; van Dyck et al., 2023). These findings represent a genuine advance in disease-modifying treatment, but they do not indicate disease arrest or reversal. Treatment involves serial administration and biomarker and imaging surveillance, while amyloid-related imaging abnormalities remain an important safety concern (Greenberg et al., 2025; Rafii and Aisen, 2025). Thus, the remaining therapeutic need is not evidence that Aβ is irrelevant, but evidence that effective intervention must account for target selection, disease stage, patient eligibility, treatment exposure and safety.

Gene therapy offers a complementary strategy by enabling sustained production, suppression or editing of disease-relevant molecules within the central nervous system. Recombinant adeno-associated virus (AAV) vectors are prominent in this field because they can transduce long-lived neural cells and can be engineered through both capsid selection and regulatory-cassette design (Hudry and Vandenberghe, 2019; Gao et al., 2024). Nevertheless, AAV is neither intrinsically precise nor immunologically inert. The therapeutic profile of an AAV product depends on the interaction among capsid, expression cassette and payload, together with dose, administration route, anatomical coverage, host immunity, species and disease state (Ling et al., 2023; Wang et al., 2024). Durable expression is therefore double-edged: it may reduce the need for repeated administration, but it can also prolong off-target or payload-related effects and is difficult to titrate, terminate or repeat.

Adeno-associated virus-mediated approaches to AD have targeted Aβ production and clearance, tau expression and propagation, APOE-associated biology, neuroinflammatory and microglial pathways, and neurotrophic or synaptic support. These interventions should not be treated as a single therapeutic class merely because they share the same vector platform: each vector–payload product has a distinct mechanism, therapeutic window and risk profile. Moreover, most strategies remain supported primarily by rodent proof-of-concept studies, with much less validation in human-derived systems, nonhuman primates or clinical trials. In the randomized AAV2-NGF trial, stereotactic delivery was feasible and generally well tolerated, but no benefit was detected on clinical outcomes or the selected AD biomarkers (Rafii et al., 2018). This experience illustrates that successful administration or durable transgene expression does not necessarily translate into clinical efficacy.

The central translational question is therefore not simply whether AAV can enter the brain or sustain expression, but whether a particular vector–payload product can achieve an appropriate exposure in the relevant cells and brain regions, at the appropriate disease stage and within an acceptable therapeutic window. This review, informed by a documented multi-database literature search, critically evaluates AAV-mediated therapeutic strategies for AD using this alignment framework. We distinguish vector performance from payload-specific biology and evaluate delivery and biodistribution, molecular target engagement, pathological modification, functional outcomes, safety and clinical efficacy as separate evidence domains. We argue that translational readiness should be judged by biological fit and evidence maturity rather than by transduction efficiency or expression durability alone.

## 2 Literature search and evidence identification

This narrative review was informed by searches of PubMed, Web of Science Core Collection and ClinicalTrials.gov conducted in July 2026, supplemented by citation tracking and targeted publication reconciliation. Search terms combined Alzheimer disease and adeno-associated virus concepts with therapeutic targets, delivery, safety, clinical development and biomarkers. Reports were eligible if they evaluated an AAV-mediated therapeutic intervention relevant to AD or provided directly relevant translational evidence; reviews, non-AAV interventions, purely descriptive modeling studies and reports without unique evidence were excluded. Records were screened at the title-and-abstract and full-report levels, and related publications or versions were linked to reduce double counting. Of 943 records screened, 374 reports underwent full-report assessment and 363 publication reports were included and linked into 196 publication-derived Study Families. Six eligible ClinicalTrials.gov records representing four clinical programme families were evaluated separately. Evidence was synthesized narratively, with preclinical and clinical evidence and distinct outcome domains considered separately. Full search strategies, eligibility criteria and record-handling procedures are provided in the Supplementary methods, Supplementary Data 1, 2 and Supplementary Figure 1.

## 3 Principles governing CNS-directed AAV delivery

### 3.1 Vector architecture and product-level specificity

Recombinant AAV vectors retain viral inverted terminal repeats while replacing viral coding sequences with therapeutic expression cassettes. Their limited packaging capacity (∼4.7 kb), episomal persistence and ability to transduce post-mitotic cells have made them attractive platforms for CNS gene delivery. However, AAV performance is not determined by capsid selection alone. Natural and engineered capsids differ in receptor interaction, intracellular processing, immunogenicity and tissue distribution (Deverman et al., 2016; Körbelin et al., 2016; Goertsen et al., 2022; Chen et al., 2023; Santoscoy et al., 2024), while the final therapeutic profile is shaped by the combined effects of capsid, regulatory elements, payload, dose and host context (Pillay et al., 2016; Dimidschstein et al., 2016; Ling et al., 2022, 2023; Gleichman et al., 2023).

Long-term expression is a major advantage of AAV in neuronal applications, but persistence alone does not guarantee therapeutic benefit. In the AAV2-NGF clinical programme for AD, prolonged transgene expression was detected years after administration, yet limited vector spread and incomplete targeting of relevant cholinergic populations highlighted the challenge of translating expression durability into functional efficacy (Castle et al., 2020). Therefore, AAV specificity should be considered a property of the complete vector–payload product rather than a simple characteristic of the capsid. Relevant factors affecting CNS targeting and translational interpretation are summarized in Supplementary Table 2.

### 3.2 Delivery route and BBB biology constrain CNS exposure

The BBB represents a major determinant of CNS gene delivery. The principal physical barrier is formed by specialized brain microvascular endothelial cells and their intercellular tight junctions, whereas pericytes, basement-membrane components and astrocytic end-feet contribute to barrier regulation within the broader neurovascular unit (Armulik et al., 2010; Daneman et al., 2010). AAV entry into the CNS involves multiple steps, including receptor interaction, endocytosis, intracellular trafficking and nuclear transport. Structural studies of AAVrh.10 have identified capsid determinants associated with brain vascular transport, supporting the concept that BBB interaction is capsid-dependent rather than a universal property of all AAV vectors (Albright et al., 2018).

Systemic AAV delivery is strongly dependent on species and host context. Intravenous AAV9 produced widespread CNS transduction in mice, but neuronal and astrocytic targeting differed between neonatal and adult animals (Foust et al., 2009). AAV-PHP.B showed strong CNS activity in C57BL/6J mice, but the enhanced phenotype was not reproduced in BALB/cJ mice or nonhuman primates (Hordeaux et al., 2018). Its biodistribution also differed markedly between mice and rhesus macaques, particularly after intravascular administration (Liguore et al., 2019). Claims of BBB crossing should therefore be interpreted in the specific context of species, age, administration route and dose rather than generalized across capsids or experimental systems.

Administration route determines both anatomical coverage and safety. Intraparenchymal injection provides high local exposure but has limited spread and requires invasive targeting. Delivery through the cerebrospinal fluid can broaden distribution across the neuraxis, but parenchymal penetration may remain uneven. Intravenous delivery may reach multiple CNS regions but usually requires greater systemic exposure. In nonhuman primates, intrathecal AAV9 produced broad but incomplete CNS transduction (Gray et al., 2013). In an AD-directed programme, intracisternal and intraventricular AAVrh.10-APOE2 both produced widespread vector and transgene distribution, but the magnitude and regional pattern varied among animals and routes (Rosenberg et al., 2018). Figure 1 summarizes how vector–payload design, administration route, BBB and CNS interfaces, regional biodistribution and cellular context jointly shape therapeutic exposure.

**FIGURE 1:**
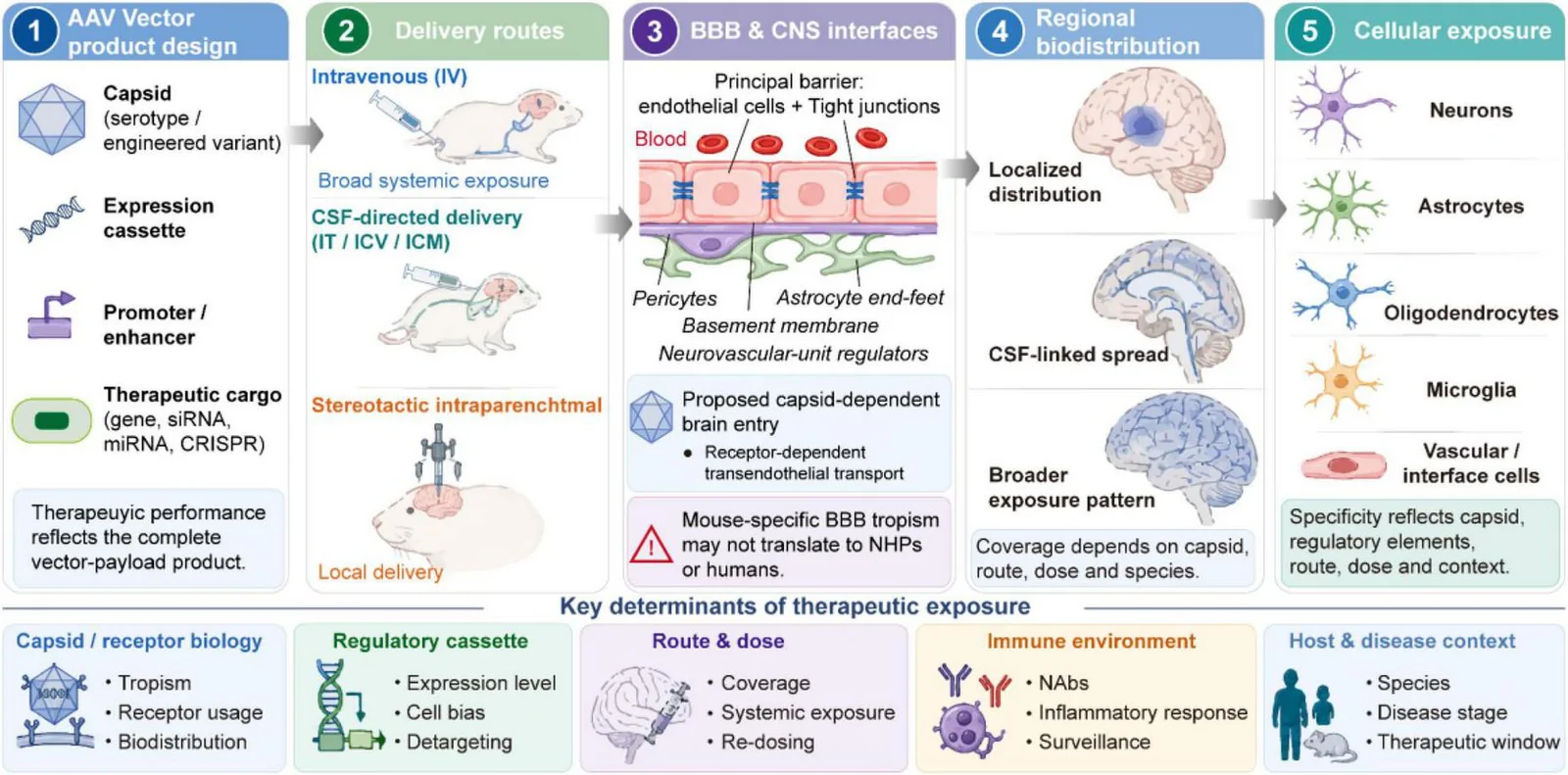
Adeno-associated virus (AAV) delivery to the CNS: administration routes, BBB interfaces, biodistribution and determinants of therapeutic exposure. AAV-mediated CNS exposure depends on vector–payload design, administration route, BBB and neurovascular interfaces, regional biodistribution and cellular context. Brain endothelial cells and tight junctions constitute the principal physical BBB, while other neurovascular-unit components regulate barrier properties. Delivery pattern and cellular exposure are capsid-, route-, dose- and host-dependent, and BBB tropism observed in mice should not be directly extrapolated to nonhuman primates or humans. AAV, adeno-associated virus; BBB, blood–brain barrier; CNS, central nervous system; CRISPR, clustered regularly interspaced short palindromic repeats; CSF, cerebrospinal fluid; ICM, intracisterna magna; ICV, intracerebroventricular; IT, intrathecal; IV, intravenous; miRNA, microRNA; NAbs, neutralizing antibodies; NHP, nonhuman primate; siRNA, small interfering RNA.

### 3.3 Translational barriers beyond transduction efficiency

Beyond delivery, immune compatibility, packaging constraints and expression control remain major challenges for CNS AAV applications. Pre-existing neutralizing antibodies can reduce vector exposure, while immune responses against capsid or transgene products may influence safety and durability (Calcedo et al., 2009). High-dose AAV administration has also raised concerns regarding tissue-specific toxicity, including dorsal root ganglion pathology observed in nonhuman primate studies (Hordeaux et al., 2020a). Suppressing transgene expression in sensory neurons through microRNA target sequences reduced this pathology, showing that safety can depend on expression-cassette design as well as capsid distribution (Hordeaux et al., 2020b).

The limited packaging capacity of AAV restricts delivery of large genes and complex editing systems. Oversized genomes can generate heterogeneous and truncated products, reducing functional expression (Wu et al., 2010). In addition, promoter selection and payload design determine expression magnitude and duration, which is particularly important for AD applications involving neurotrophic factors, immune modulators or gene-editing approaches.

Therefore, AAV selection for AD should not be guided solely by maximal transduction efficiency. Successful translation requires alignment among vector design, delivery route, target-cell exposure, disease stage and clinically meaningful outcomes (Gao et al., 2024). The major translational requirements and corresponding development priorities are summarized in Table 1.

**TABLE 1:** Key translational requirements for AAV-based therapies in Alzheimer’s disease.

Translational consideration	Central issue in AD	Priority for development	Balanced interpretation	References
Delivery and brain coverage	Capsid performance varies across species, routes and doses, and focal expression may not cover distributed AD-relevant circuits	Quantify dose, regional biodistribution and target-cell coverage in human-relevant models and NHPs	Strong rodent transduction does not establish human applicability; limited efficacy may reflect inadequate exposure rather than payload failure	Hordeaux et al., 2018; Liguore et al., 2019; Castle et al., 2020; Hogestyn et al., 2025
Durability, immunity and re-dosing	Persistent expression may prolong both benefit and adverse effects; pre-existing or treatment-induced immunity can restrict dosing and re-administration	Characterize long-term expression, immune responses and feasibility of regulating or repeating treatment	Durability is advantageous only when sustained payload exposure remains appropriate and safe	Calcedo et al., 2009; Hinderer et al., 2018; Hordeaux et al., 2020b; Xu et al., 2025
Payload biology and safety	Toxicity may arise from the payload as well as the vector, and the same pathway can produce different effects across disease contexts	Separate vector-related from payload-related risks and assess dose, cell type and disease-stage dependence	Target engagement does not establish therapeutic benefit; a biologically active payload may be beneficial or harmful depending on context	Kou et al., 2011; Kiyota et al., 2012; Chakrabarty et al., 2015; Rafii et al., 2018
Model relevance and evidence maturity	Rodent efficacy, NHP delivery, biomarker changes and randomized clinical outcomes answer different questions	Use human-derived systems and NHPs for translational bridging and distinguish target engagement from functional and clinical efficacy	Positive preclinical findings should not be directly extrapolated to patients, while negative clinical results may also reveal delivery or target-selection limitations	Castle et al., 2020; Drouyer et al., 2024; Johnson et al., 2025
Patient stratification and clinical endpoints	AD heterogeneity may alter payload relevance according to genotype, pathology and disease stage	Match enrollment to payload biology and combine target-engagement biomarkers with cognitive, functional and safety outcomes	Biomarkers can improve patient selection and mechanistic interpretation but cannot substitute for clinically meaningful benefit	Rafii et al., 2018; Jack et al., 2024; Johnson et al., 2025
Manufacturing and regulatory readiness	Product purity, genome integrity, capsid composition and potency affect effective exposure and clinical reproducibility	Define critical quality attributes and establish comparable potency across research, toxicology and clinical-grade material	Manufacturing quality cannot rescue an ineffective payload, but biological efficacy alone is insufficient without a reproducible clinical product	Ohba et al., 2023; McColl-Carboni et al., 2024

These domains represent interdependent translational requirements rather than absolute barriers. Their importance varies with vector, payload, administration route, disease stage and intended patient population. Representative therapeutic and clinical study-level evidence is detailed in Supplementary Table 1. AAV, adeno-associated virus; AD, Alzheimer’s disease; APOE, apolipoprotein E; CNS, central nervous system; NHP, nonhuman primate.

## 4 AAV-mediated therapeutic strategies for Alzheimer’s disease

Alzheimer’s disease involves interacting amyloid, tau, genetic, immune and synaptic processes, and AAV should therefore be viewed as a delivery platform for mechanistically distinct payloads rather than a uniform therapeutic modality. Current strategies target amyloid biology, APOE-associated risk, tau pathology, neuroimmune pathways, and neurotrophic or synaptic support (Knopman et al., 2021). Figure 2 summarizes these therapeutic domains and their relative evidence maturity. Most approaches remain supported primarily by preclinical studies, with uneven validation in human-derived systems, nonhuman primates and clinical trials. Accordingly, delivery and expression, target engagement, pathological modification, functional benefit and clinical efficacy are treated as distinct evidence domains. Representative evidence is compared in Table 2, and clinical programmes are summarized separately in Box 1.

**FIGURE 2:**
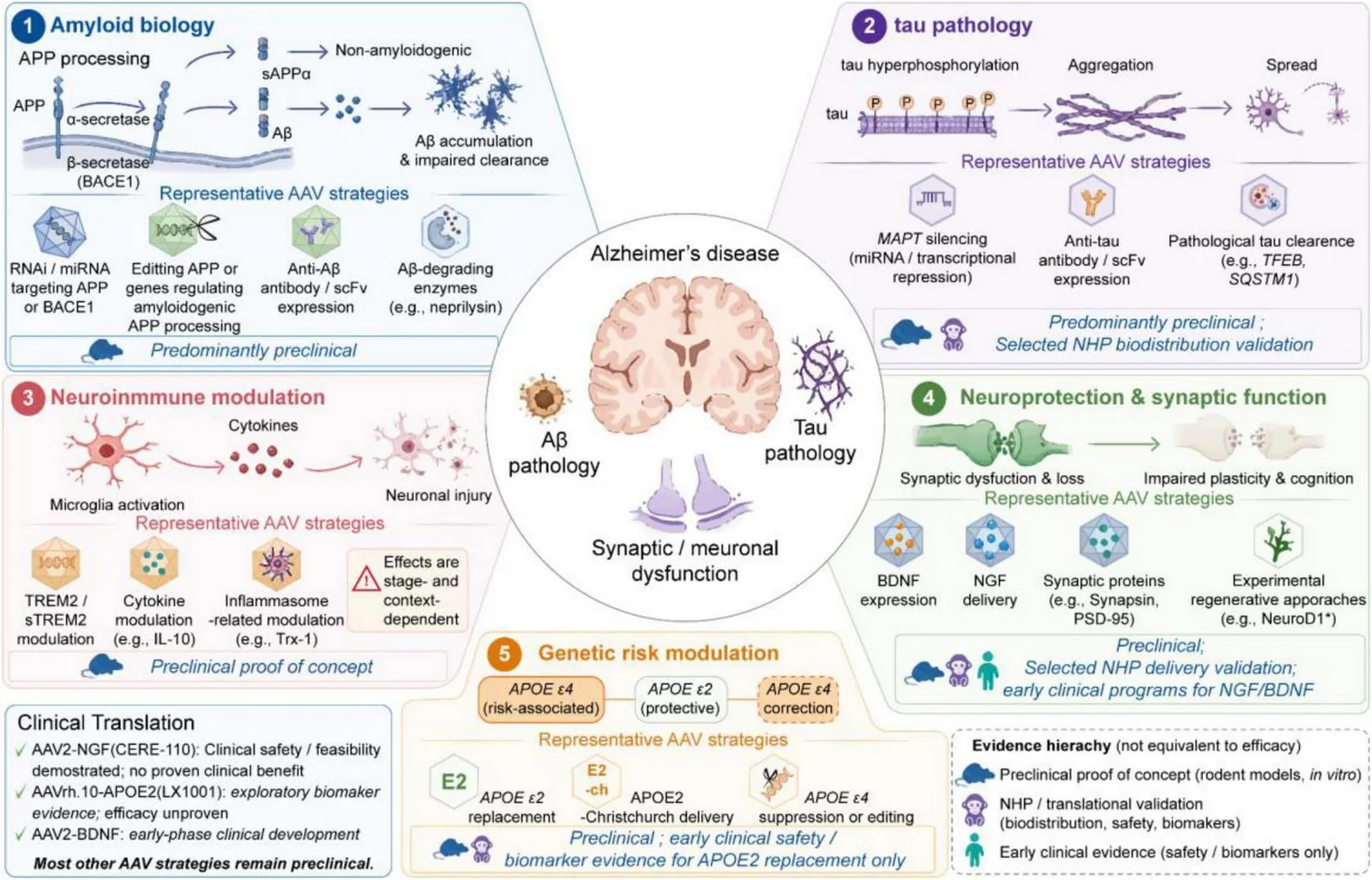
Representative AAV-based therapeutic strategies for Alzheimer’s disease and their evidence maturity. AAV-based approaches target amyloid biology, tau pathology, neuroimmune pathways, neurotrophic and synaptic support, and APOE-associated genetic risk. Most remain preclinical, whereas selected programmes have additional nonhuman-primate or early clinical evidence. These higher evidence levels primarily reflect biodistribution, safety, target engagement or biomarker findings rather than established therapeutic efficacy. *NeuroD1-associated functional improvement does not establish bona fide astrocyte-to-neuron conversion; its lineage specificity and mechanism remain unresolved. AAV, adeno-associated virus; Aβ, amyloid-β; APOE, apolipoprotein E; APP, amyloid precursor protein; BACE1, β-site APP-cleaving enzyme 1; BDNF, brain-derived neurotrophic factor; CSF, cerebrospinal fluid; IL-10, interleukin-10; MAPT, microtubule-associated protein tau; miRNA, microRNA; NGF, nerve growth factor; NHP, nonhuman primate; scFv, single-chain variable fragment; PSD-95, postsynaptic density protein 95; RNAi, RNA interference; sAPPα, soluble amyloid precursor protein alpha; SQSTM1, sequestosome 1; sTREM2, soluble triggering receptor expressed on myeloid cells 2; TFEB, transcription factor EB; TREM2, triggering receptor expressed on myeloid cells 2; Trx-1, thioredoxin-1.

**TABLE 2:** Representative preclinical evidence for AAV-mediated therapeutic strategies in Alzheimer’s disease.

Therapeutic domain	Representative AAV approaches and models	Main evidence	Key evidence boundary	References
Aβ production and clearance	APP RNAi, anti-BACE1 VHH, neprilysin and anti-Aβ scFv; AD mouse models	Reduced Aβ production or burden and improved selected pathological, synaptic or cognitive outcomes	Effects depend on Aβ species, target selectivity and CNS coverage; sustained expression may introduce vascular or on-target risks	Rodríguez-Lebrón et al., 2009; Kou et al., 2011; Iwata et al., 2013; Marino et al., 2022; Selles et al., 2023
APOE risk modification	*APOE* ε2 / APOE2-Christchurch supplementation, APOE4 suppression or editing; humanized APOE mice with selected NHP validation	Reduced amyloid-related pathology; APOE2-Christchurch also modified tau pathology; editing achieved molecular *APOE* ε4 correction	Replacement, suppression and editing are distinct strategies; long-term safety and functional efficacy remain incomplete	Zhao et al., 2016; Rosenberg et al., 2018; Günaydin et al., 2024; Jackson et al., 2024; Karan et al., 2024; Günaydin et al., 2026
tau-directed strategies	MAPT repression/miRNA, TFEB/SQSTM1-mediated clearance and vectored anti-tau antibodies; tauopathy mice with NHP validation	Reduced tau expression or pathology and improved selected neuronal or functional outcomes	NHP data highlight biodistribution limits; sustained tau lowering and antibody exposure require dose and safety control	Polito et al., 2014; Ising et al., 2017; Xu et al., 2019; Wegmann et al., 2021; Hogestyn et al., 2025
Neuroimmune modulation	TREM2/sTREM2, IL-10, Trx-1 and adiponectin; APP/PS1 and 5xFAD models	Modified microglial, cytokine or inflammasome pathways with context-dependent pathological and behavioral effects	Effects vary with disease stage, payload and cell state; anti-inflammatory activity does not uniformly predict benefit	Kiyota et al., 2012; Chakrabarty et al., 2015; Sheng et al., 2019; Zhong et al., 2019; Ng et al., 2024; Jia et al., 2025
Neurotrophic support	AAV-BDNF in tauopathy and AD mouse models with NHP delivery validation	Improved neuronal survival, synaptic integrity and cognition, sometimes without major Aβ or tau reduction	NHP studies mainly validate delivery; human therapeutic efficacy remains unproven	Jiao et al., 2016; Nagahara et al., 2018; Tang et al., 2026
NeuroD1-associated regenerative strategies	AAV-NeuroD1 in AD mouse models and an induced hTau AD-like NHP model, with independent lineage-tracing studies	Reported selected neuronal, structural, metabolic and functional improvements	Functional benefit does not prove mature astrocyte-to-neuron conversion; NHP evidence from an induced hTau model remains preliminary; lineage specificity and long-term safety remain unresolved	Xie et al., 2023; Chen et al., 2025; Wu et al., 2025; Jiang et al., 2026

Selected studies illustrate representative preclinical AAV strategies and their main evidence boundaries rather than provide a comprehensive ranking of therapeutic potential. Human clinical programmes are summarized separately in Box 1, and detailed study-level information is provided in Supplementary Table 1. AAV, adeno-associated virus; Aβ, amyloid-β; AD, Alzheimer’s disease; APOE, apolipoprotein E; APP, amyloid precursor protein; BACE1, β-site amyloid precursor protein cleaving enzyme 1; BDNF, brain-derived neurotrophic factor; CNS, central nervous system; IL-10, interleukin-10; MAPT, microtubule-associated protein tau; miRNA, microRNA; NHP, nonhuman primate; scFv, single-chain variable fragment; RNAi, RNA interference; SQSTM1, sequestosome 1; sTREM2, soluble triggering receptor expressed on myeloid cells 2; TFEB, transcription factor EB; TREM2, triggering receptor expressed on myeloid cells 2; Trx-1, thioredoxin-1; VHH, variable domain of heavy-chain-only antibody.

**BOX 1:** Clinical development of AAV-based therapies for Alzheimer’s disease.

Status date: An audited ClinicalTrials.gov search performed on 25 July 2026 identified six eligible registry records representing four clinical development programmes. Registry records, extension studies and linked publications were consolidated at the programme level to avoid double counting.				
**Programme**	**Development status**	**Vector and delivery**	**Primary objective and evidence to date**	**Key records**
CERE-110 / AAV2-NGF	Phase I completed, *n* = 10; randomized sham-controlled Phase II completed, *n* = 49	Bilateral stereotactic AAV2-NGF delivery to the basal forebrain; Dose: Phase II total 2.0 × 10^11 vg, dose-escalation in Phase I	Durable NGF expression and biological activity were demonstrated, but Phase II showed no significant clinical benefit. Postmortem analysis identified limited vector spread and incomplete target coverage	NCT00087789 (Clinical Trials.gov., 2004); NCT00876863 (Clinical Trials.gov., 2009; Rafii et al., 2014, 2018; Castle et al., 2020)
LX1001 / AAVrh.10-APOE2	Open-label Phase I/II completed, *n* = 15; long-term follow-up ongoing	Single CSF administration at the craniocervical junction; C1, 1.4 × 10^10 gc/mL CSF; C2, 4.4 × 10^10 gc/mL CSF; C3, 1.4 × 10^11 gc/mL CSF; C4, 1.4 × 10^14 gc fixed dose (≈3.4 × 10^11 gc/mL CSF).	Preliminary conference-level evidence supports tolerability, dose-dependent CSF ApoE2 protein expression and exploratory biomarker changes; cognitive efficacy and disease modification remain unproven	NCT03634007 (Clinical Trials.gov., 2018); NCT05400330 (Clinical Trials.gov., 2022; Johnson et al., 2025)
AAV2-BDNF	Phase I, active, not recruiting; planned *n* = 12 in early AD or MCI	MRI-guided intraparenchymal delivery to the entorhinal cortex; exact clinical vector dose NR in the available conference report/registry materials	Conference-level interim data support procedural feasibility and regional FDG-PET increases in a small treated subset; clinical efficacy has not been established	NCT05040217 (Clinical Trials.gov., 2021; Tuszynski et al., 2025)
AAV-hTERT	Phase I registry record; status unknown; planned *n* = 5	Single IV plus IT AAV-hTERT administration; capsid serotype and exact dose not publicly specified	No results have been posted. The programme is retained for completeness but currently provides no evaluable clinical evidence	NCT04133454 (Clinical Trials.gov., 2019)

Clinical perspective. ClinicalTrials.gov records were consolidated with extension studies and linked publications at the programme level to avoid double counting. Trial status reflects the registry information available at the final clinical-trial audit. Conference or supplement reports are identified as preliminary evidence and are not considered equivalent to full peer-reviewed efficacy reports. Detailed dose, endpoint, safety and evidence-boundary information for these programmes is provided in Supplementary Table 1. AAV, adeno-associated virus; AD, Alzheimer’s disease; APOE, apolipoprotein E; BDNF, brain-derived neurotrophic factor; CSF, cerebrospinal fluid; FDG-PET, fluorodeoxyglucose positron emission tomography; gc, genome copies; hTERT, human telomerase reverse transcriptase; IT, intrathecal; IV, intravenous; MCI, mild cognitive impairment; MRI, magnetic resonance imaging; NR, not reported; NGF, nerve growth factor; vg, vector genomes.

### 4.1 Targeting amyloid biology

Amyloid-β remains a major therapeutic target in AD, but amyloid reduction should not be equated with complete disease control. In phase III trials, lecanemab and donanemab produced substantial amyloid lowering and slowed clinical decline in early symptomatic AD, while disease progression continued and amyloid-related imaging abnormalities remained an important safety consideration (Sims et al., 2023; van Dyck et al., 2023). These clinical observations provide an important benchmark for AAV approaches, which have primarily sought either to reduce Aβ production or to enhance its clearance and neutralization.

Reducing amyloidogenic processing was among the earliest AAV strategies. Allele-specific rAAV-mediated RNA interference against the Swedish APP variant selectively reduced mutant APP and soluble Aβ in APP/PS1 mice and attenuated behavioral progression, despite little effect on plaque number (Rodríguez-Lebrón et al., 2009). A more recent approach used a BBB-crossing AAV vector to express a BACE1-selective VHH. A single systemic administration produced sustained VHH expression and improved amyloid burden, neuroinflammation, synaptic measures and cognitive performance in *App*NL-G-F mice (Marino et al., 2022). These studies also illustrate an important distinction for RNA-based therapy. AAV can provide sustained expression of an RNAi payload, but it does not remove the biological risks of prolonged target suppression. Sequence selectivity, dose, and preservation of the physiological functions of APP and BACE1 therefore remain product-specific constraints.

A complementary strategy is to increase Aβ degradation or neutralize toxic Aβ species. Systemic AAV-mediated neprilysin delivery achieved broad neuronal expression, reduced Aβ oligomers and amyloid burden, and improved learning and memory in an AD mouse model (Iwata et al., 2013). Antibody-based gene delivery has increasingly moved toward species-selective targeting. AAV9-mediated neuronal expression of the oligomer-selective single-chain variable fragment (scFv) NUsc1 protected synapses and reversed memory deficits in aged APPswe/PS1ΔE9 mice; the same construct also produced secreted antibody in adult human brain slices (Selles et al., 2023). These findings support targeting biologically active Aβ species rather than relying only on total plaque burden as a therapeutic readout.

Sustained anti-Aβ expression may nevertheless introduce liabilities that differ from intermittent pharmacological treatment. In an earlier AAV-scFv study, reduced parenchymal amyloid was accompanied by focal increases in vascular amyloid and cerebral hemorrhage, illustrating that successful Aβ mobilization does not necessarily imply improved safety (Kou et al., 2011). This issue is particularly relevant to AAV because transgene exposure cannot be readily withdrawn once durable CNS expression has been established. Thus, the translational challenge for amyloid-directed AAV therapy is not simply to maximize Aβ lowering. It is to match the therapeutic payload with the relevant Aβ species and disease stage, achieve sufficient anatomical coverage at an acceptable dose, and demonstrate that pathological target engagement translates into functional benefit without unacceptable cerebrovascular or vector-related toxicity.

### 4.2 Modulating APOE-associated genetic risk

Apolipoprotein E-directed therapy is better considered a genetic-risk strategy than a subtype of amyloid clearance. *APOE ε4* is the strongest common genetic risk factor for late-onset AD, whereas *APOE ε2* is associated with substantial protection (Corder et al., 1993, 1994). APOE manipulation can also influence plaque-associated microglial responses, synaptic injury and tau pathology (Günaydin et al., 2024; Jackson et al., 2024). Its therapeutic effects are therefore likely to depend on APOE isoform, target cell, expression level and disease context.

*APOE* ε2 gene supplementation is the most developed preclinical approach. Intracerebral AAV-mediated ApoE2 expression reduced soluble and insoluble Aβ and amyloid deposition in mouse models, with efficacy depending on ApoE2 expression and pre-existing pathology (Zhao et al., 2016). More recently, ependymal AAV-mediated ApoE2 expression reduced amyloid deposition, plaque-associated microglial activation and synaptic loss despite continued ApoE4 expression, although cognition and comprehensive toxicology were not evaluated (Jackson et al., 2024). AAVrh.10-mediated APOE2 delivery was also evaluated in nonhuman primates for CNS distribution and preclinical safety (Rosenberg et al., 2018). Together, these studies support APOE2 replacement, while also showing that different capsids, routes and target cells cannot be treated as interchangeable therapeutic products.

More selective strategies aim to modify the APOE payload or reduce the risk allele itself. AAVrh.10-mediated APOE2-Christchurch expression reduced amyloid pathology similarly to unmodified APOE2. In the tested *APOE* ε4-background tau model, it also reduced tau pathology and glial activation (Günaydin et al., 2024). AAV.S2-delivered miRNAs achieved marked *APOE* ε4 suppression in targeted brain regions (Karan et al., 2024), while a split-intein dual-AAV prime-editing system converted the APOE ε4 allele to ε3 in humanized TRE4 mice (Günaydin et al., 2026). The editing study demonstrated molecular feasibility but did not assess AD pathology, cognition or long-term safety. Greater genetic specificity may improve biological matching, but also introduces additional questions about editing efficiency, off-target effects, dual-vector delivery and irreversible modification.

*APOE* ε2 gene replacement has now reached early human evaluation through LX1001. The completed open-label Phase 1/2 study NCT03634007 enrolled 15 *APOE* ε4/ε4 participants receiving AAVrh.10-hAPOE2. A 2025 conference abstract reported generally acceptable tolerability, dose-dependent CSF ApoE2 protein expression and exploratory amyloid and tau biomarker changes, with no amyloid-related imaging abnormalities reported during the available follow-up (Johnson et al., 2025). Long-term safety is being assessed in NCT05400330. However, the available Johnson report is a conference/supplement abstract rather than a full efficacy publication, and cognitive benefit or disease modification has not been established. LX1001 therefore provides important evidence of genotype-defined clinical feasibility and target engagement, but not yet validated therapeutic efficacy. Clinical programme details are summarized in Box 1, with representative APOE-directed evidence compared in Table 2.

### 4.3 Targeting tau pathology

Adeno-associated virus strategies directed against tau have a therapeutic logic distinct from amyloid-oriented approaches. They aim to reduce tau production, enhance the clearance of pathological tau, or provide sustained neutralization of disease-associated tau species. Because tau also has physiological neuronal functions, sustained intervention requires control of both the magnitude and anatomical distribution of target modulation. Current evidence remains predominantly preclinical, although recent studies have begun to test whether tau-lowering payloads retain activity in nonhuman primates.

Sustained reduction of MAPT expression is among the most developed AAV-based tau strategies. AAV-delivered zinc-finger transcriptional repressors produced prolonged tau reduction in the adult mouse brain and reduced amyloid-associated neuritic injury in APP/PS1 mice (Wegmann et al., 2021). More recently, AAV delivery of the artificial miRNA amiR^Tau01 reduced pathological phosphorylated tau in Tau22 mice. In nonhuman primates, the same payload produced clear MAPT knockdown in individual transduced neurons, but insufficient brain biodistribution prevented measurable reduction at the bulk-tissue level (Hogestyn et al., 2025). These findings distinguish payload potency from therapeutic exposure and show why successful target engagement in individual cells does not ensure adequate brain-wide efficacy.

A second strategy is to enhance the intracellular clearance of pathological tau. AAV-mediated TFEB expression reduced abnormal tau accumulation and neurotoxicity in tauopathy mice, supporting lysosomal enhancement as one route to tau clearance (Polito et al., 2014). More selective cargo recognition has also been explored. SQSTM1 preferentially targeted pathological tau species and reduced neurofibrillary pathology and tau spreading *in vivo* (Xu et al., 2019). These mechanisms should not be treated as equivalent. Broad lysosomal activation and selective recognition of pathological cargo differ in both biological scope and potential safety consequences.

Adeno-associated virus-vectored anti-tau antibodies provide a third approach. Local AAV-mediated expression of anti-tau scFvs reduced soluble and phosphorylated tau in P301S mice (Ising et al., 2017), while intracerebral AAVrh.10-mediated expression of the PHF1 antibody markedly reduced regional insoluble tau and neurofibrillary pathology (Liu et al., 2016). More recently, intramuscular AAV1 delivery of an MC1-derived scFv reduced several cerebral tau species and improved selected functional outcomes in P301S mice, whereas combining MC1- and PHF1-derived scFvs did not provide additive benefit (Katel et al., 2025). These studies establish proof of concept for sustained vectorized tau immunotherapy, but remain rodent evidence. Overall, tau-directed AAV therapy is biologically compelling, particularly when tau pathology is established, but translation will require adequate CNS coverage, controlled knockdown or antibody exposure, and long-term neuronal safety.

### 4.4 Modulating neuroimmune function and inflammatory signaling

Neuroimmune modulation should not be equated with generalized suppression of inflammation. Microglia can contribute to amyloid containment and clearance, but may also promote synaptic injury under particular disease conditions. This context dependence is especially relevant to AAV because prolonged payload expression may extend beyond the disease stage in which a given immune response is beneficial. TREM2 and cytokine-directed studies provide direct experimental examples of this problem.

Triggering receptor expressed on myeloid cells 2 illustrates the stage- and payload-dependent nature of microglial intervention. In APP/PS1 mice, TREM2-dependent microglial activity promoted synaptic loss at earlier stages but contributed to amyloid control later in disease (Sheng et al., 2019). By contrast, AAV-mediated expression of soluble TREM2 in 5xFAD mice enhanced selected microglial responses and improved amyloid, synaptic and behavioral outcomes (Zhong et al., 2019). Full-length TREM2, soluble TREM2 and loss-of-function manipulations therefore cannot be interpreted as interchangeable interventions, and induction of a disease-associated microglial state should not by itself be considered evidence of neuroprotection.

Cytokine delivery provides an even clearer example of context-dependent immune modulation. Hippocampal AAV2/1-mediated IL-10 expression reduced gliosis and improved neurogenesis and spatial learning in APP+PS1 mice without directly targeting amyloid pathology (Kiyota et al., 2012). In contrast, AAV2/1-mediated IL-10 expression in other APP models increased amyloid accumulation, impaired glial Aβ phagocytosis and worsened memory (Chakrabarty et al., 2015). Thus, classification of a cytokine as anti-inflammatory does not predict its therapeutic effect. Cell type, disease stage, spatial exposure and preservation of beneficial microglial functions are likely to be more informative.

Inflammasome-directed strategies offer a more pathway-focused alternative. Liver-directed AAV2/8 adiponectin expression reduced microglial NLRP3 inflammasome activation, amyloid pathology and cognitive deficits in 5xFAD mice, showing that peripheral AAV delivery can modify CNS immune signaling without direct CNS vector delivery (Ng et al., 2024). AAV-mediated thioredoxin-1 expression has also been associated with reduced TXNIP-NLRP3 signaling and pyroptosis-related readouts in APP/PS1 models (Jia et al., 2025). However, NLRP3 activation, caspase-1-dependent cytokine maturation and terminal gasdermin-mediated pyroptosis are distinct processes. The latter study is therefore best interpreted as pathway-level mechanistic evidence rather than proof that modulation of a single inflammasome component is sufficient to control the full pyroptotic programme. Overall, neuroimmune AAV therapy will require stage- and cell-state-specific modulation that preserves beneficial immune functions rather than sustained nonspecific inflammatory suppression.

### 4.5 Neurotrophic and regenerative strategies

Neurotrophic strategies differ from amyloid- and tau-directed approaches because their primary aim is to preserve or restore vulnerable neural circuits rather than remove a defining pathological protein. Functional benefit may therefore occur without major changes in amyloid or tau. At the same time, durable growth-factor expression places particular demands on anatomical targeting, exposure control and long-term safety (Rafii et al., 2014). However, a subsequent randomized Phase II trial involving 49 patients found no significant benefit on clinical outcomes or selected AD biomarkers despite acceptable tolerability (Rafii et al., 2018). Postmortem analysis later showed NGF expression persisting for up to 7 years, but vector spread was limited and the intended cholinergic neurons were not directly engaged at the examined injection sites (Castle et al., 2020). These findings neither establish failure of NGF biology nor support clinical efficacy. Instead, they show that durable expression, anatomical coverage and therapeutic benefit are distinct outcome levels.

Brain-derived neurotrophic factor provides a complementary function-preserving strategy. In P301L tauopathy mice, intracerebroventricular AAV-BDNF improved behavioral performance, neuronal survival and synaptic integrity without reducing tau hyperphosphorylation, supporting neuroprotection that can occur independently of direct pathological clearance (Jiao et al., 2016). MRI-guided AAV2-BDNF delivery to the entorhinal cortex of nonhuman primates subsequently demonstrated accurate targeting, neuronal transduction and distribution toward hippocampal circuitry (Nagahara et al., 2018). Hippocampus-targeted AAVT42-BDNF improved selected neuronal and behavioral/cognitive outcomes in APP/PS1, rTg4510 and 3 × Tg mouse models (Tang et al., 2026). AAV2-BDNF has also entered Phase I clinical evaluation in early AD and mild cognitive impairment (ClinicalTrials.gov NCT05040217), but clinical efficacy has not been established. Together, these studies support the biological and delivery rationale for BDNF while leaving human safety and therapeutic benefit to be determined. Clinical details for AAV2-NGF and AAV2-BDNF are summarized in Box 1.

NeuroD1-based approaches extend this concept toward regenerative intervention, but their mechanism requires careful qualification. Brain-wide AAV-NeuroD1 delivery improved neuronal and cognitive outcomes in 5xFAD mice, and a more recent study reported reduced neuronal damage, hippocampal atrophy and neuroinflammation together with improved glucose metabolism and spatial working memory in an experimentally induced hTau overexpression AD-like nonhuman-primate model (Wu et al., 2025; Jiang et al., 2026). Given the induced model and limited cohort size, these findings should be considered preliminary translational evidence rather than validation of efficacy in sporadic AD.

However, functional improvement does not resolve the mechanism of cell replacement. Stringent lineage-tracing studies have shown that neuronal AAV leakage and labeling of pre-existing neurons can be mistaken for astrocyte-to-neuron conversion, and improved AAV tools have failed to detect robust NeuroD1-mediated conversion under several experimental conditions (Wang et al., 2021; Xie et al., 2023). More recent inducible lineage tracing provides a more nuanced picture. NeuroD1-mediated astrocyte-to-neuron conversion was detected within restricted injury contexts and time windows, but the resulting neuronal-like cells lacked mature electrophysiological properties (Chen et al., 2025). Functional improvement in AD models should therefore not be treated as evidence that widespread mature astrocyte-to-neuron conversion is the therapeutic mechanism. NeuroD1 remains an intriguing experimental strategy, but its lineage specificity, mechanism and long-term safety require further resolution.

## 5 Translational requirements and strategic priorities

Adeno-associated virus development for AD should not be judged primarily by CNS transduction alone. The more relevant question is whether a defined vector–payload product can achieve sufficient exposure in the required brain regions at a tolerable dose, produce the intended biological effect in an appropriate patient population, and ultimately generate clinically meaningful benefit. Table 1 summarizes the major translational requirements and corresponding development priorities, while Box 1 provides the current clinical programme context.

### 5.1 Dose, biodistribution and cross-species predictability

Dose and biodistribution should be treated as linked determinants of therapeutic exposure rather than as technical reporting variables. The marked CNS tropism of AAV-PHP.B was restricted to particular mouse genetic backgrounds and was not reproduced in nonhuman primates, illustrating how rodent performance can overestimate translational potential (Hordeaux et al., 2018; Liguore et al., 2019). The same principle is evident within AD programmes. Postmortem analysis of AAV2-NGF showed durable expression but a mean vector spread of only about 1 mm and incomplete engagement of the intended cholinergic targets, whereas AAV-delivered amiR^Tau01 produced MAPT knockdown in individual nonhuman-primate neurons but insufficient brain distribution to reduce MAPT at the bulk-tissue level (Castle et al., 2020; Hogestyn et al., 2025).

Cross-species validation should therefore begin before clinical dose selection rather than after a lead capsid has been optimized exclusively in rodents. Comparative screening in human iPSC-derived brain organoids identified AAV variants whose relative CNS transduction showed preliminary cross-species concordance in subsequent mouse and limited nonhuman-primate evaluations after CSF delivery (Drouyer et al., 2024). Such systems cannot replace *in vivo* pharmacology or toxicology, but they provide a practical bridge for rejecting vectors with poor human relevance. Future studies should report vector dose, administration route, regional distribution and target-cell coverage together, with head-to-head evaluation in human-relevant systems and nonhuman primates before clinical advancement. These requirements are summarized in Table 1.

### 5.2 Durability, immunity and product-specific safety

Durable expression is useful only when continued exposure remains biologically appropriate and safe. Pre-existing neutralizing antibodies against several AAV serotypes are common in humans and can restrict effective vector administration (Calcedo et al., 2009). High systemic doses have produced severe toxicities in large-animal studies, and sensory-neuron pathology in dorsal root ganglia has emerged across several CNS-directed AAV programmes (Hinderer et al., 2018; Hordeaux et al., 2020b). Importantly, transgene detargeting from sensory neurons reduced dorsal-root-ganglion toxicity in nonhuman primates, showing that some adverse effects can be mitigated through product design rather than capsid selection alone (Hordeaux et al., 2020b).

Complement-mediated toxicity and peripheral organ injury are additional concerns: systemic AAV9 has been associated with anti-capsid-antibody-dependent thrombotic microangiopathy and complement activation, while liver injury has been observed after both intravenous and intrathecal scAAV9 delivery (Hudry et al., 2023; Salabarria et al., 2024). Vector shedding also warrants route- and product-specific assessment because vector DNA may be transiently detected after CNS AAV administration without evidence of infectious particles (Krause et al., 2024); whether age- and disease-associated comorbidity modifies these risks in older adults with AD remains uncertain.

Re-dosing also cannot be assumed to remain feasible after CNS administration. Recent experimental work showed that intracranial AAV exposure can recruit B-cell responses in a dose-dependent manner and impair subsequent transduction (Xu et al., 2025). Payload biology creates an additional and separate safety layer. In AD models, sustained IL-10 expression produced beneficial outcomes in one experimental context but worsened amyloid accumulation and memory in another, while an AAV-encoded anti-Aβ scFv reduced parenchymal amyloid yet increased focal vascular amyloid and hemorrhage (Kou et al., 2011; Kiyota et al., 2012; Chakrabarty et al., 2015). Safety assessment should therefore separate capsid-related exposure from payload-related on-target and off-target effects, particularly when expression is difficult to reverse.

### 5.3 Patient stratification and clinically meaningful outcome assessment

Alzheimer’s disease heterogeneity argues against a uniform AAV treatment population. Patient selection should follow the biology of the payload. APOE-directed therapy has a clear genotype-defined population, whereas amyloid-directed approaches require confirmation of relevant amyloid pathology and tau-directed strategies may require information on tau burden and disease stage. Current biological staging frameworks support amyloid PET, validated CSF biomarkers and accurate plasma biomarkers such as p-tau217 for establishing AD pathology, while tau PET and additional biofluid markers can refine disease staging and prognosis (Jack et al., 2024). Recent cohort data further support plasma p-tau217 for pathological stratification, with GFAP and NfL providing complementary information on glial and neuronal injury rather than payload-specific target engagement (Pan et al., 2025).

Clinical trials should likewise separate target engagement from therapeutic efficacy. CSF ApoE2 protein expression and exploratory amyloid or tau biomarker changes after LX1001 provide evidence that the payload reached its intended biological compartment, but the available report is a conference-level early clinical dataset and does not establish cognitive efficacy (Johnson et al., 2025). Conversely, the randomized AAV2-NGF trial showed that acceptable tolerability and durable biological activity were not accompanied by significant clinical benefit (Rafii et al., 2018). Future trials should therefore combine product-specific target-engagement measures with AD biomarkers, cognitive and functional outcomes, imaging-based safety assessments and long-term surveillance. Rational combinations with anti-amyloid or tau-directed therapies may be considered when mechanisms are complementary, but should be tested rather than assumed to provide additive benefit.

### 5.4 Manufacturing and development readiness

Manufacturing quality is part of therapeutic exposure rather than a downstream technical issue. Recombinant AAV preparations contain full, partially packaged and empty capsids. Experimental fractionation studies showed that intermediate capsids can contribute to measured vector-genome titre and infectivity without contributing equivalent biological potency, while increasing total capsid exposure (McColl-Carboni et al., 2024). Production-system optimization can increase yield while reducing empty capsids without necessarily compromising vector function (Ohba et al., 2023). These findings support routine characterization of genome integrity, full-to-empty capsid composition and product-specific potency rather than relying on vector-genome titre alone.

A clinically credible AAV programme therefore requires comparability across research, toxicology and clinical-grade material, together with reproducible potency assays and predefined critical quality attributes. These requirements become more important as broader CNS coverage increases the total vector demand. As vector demand increases, manufacturing burden and per-dose cost become increasingly relevant, while clinical development requires predefined release specifications and comparability for product identity, genome integrity, capsid composition, purity and potency (Park et al., 2026). For AD, candidate selection should therefore consider manufacturability together with biological activity, human-relevant biodistribution and safety rather than selecting the vector with the highest rodent transduction alone. These interdependent requirements are incorporated into Table 1. Recent developments in ALS provide a broader perspective. The scaling back of some AAV programmes has drawn attention to the manufacturing, commercial and infrastructural constraints that can prevent promising gene therapies from advancing beyond early development (Liu et al., 2026).

### 5.5 Limitations of the evidence base and review approach

This review has several limitations. It is a narrative review informed by a documented multi-database search rather than a formal systematic review or meta-analysis, and no formal risk-of-bias or certainty-of-evidence assessment was performed. The search and reconciliation process identified 363 included publication reports linked into 196 publication-derived Study Families, improving evidence traceability and reducing the risk of treating related reports as independent replication. However, the evidence base remains dominated by preclinical studies and is heterogeneous in vector design, dose, route, disease model, treatment stage and outcome assessment. Rodent efficacy, nonhuman-primate delivery studies, biomarker changes and early clinical observations therefore provide different levels of translational inference and should not be interpreted as equivalent evidence of therapeutic efficacy. Some translational-support evidence on immunity, biodistribution and manufacturing also derives from non-AD settings and may not fully predict safety or performance in older patients with AD. Publication bias, incomplete reporting of negative or safety findings and the limited number of controlled human studies further constrain interpretation. These limitations preclude formal comparative ranking of therapeutic strategies but still allow identification of recurring translational requirements across AAV-based approaches.

## 6 Conclusion

Adeno-associated virus provides a versatile platform for sustained modulation of amyloid biology, APOE-associated genetic risk, tau pathology, neuroimmune pathways and neurotrophic support in AD. However, AAV is neither intrinsically precise nor intrinsically disease modifying. Its therapeutic value is determined by the complete vector–payload product and by its fit with patient biology, disease stage, anatomical coverage and dose. Current studies demonstrate that CNS delivery, durable transgene expression and pathological target engagement are achievable, but human evidence also shows that these outcomes do not necessarily translate into cognitive benefit (Rafii et al., 2018; Castle et al., 2020; Johnson et al., 2025).

Non-viral and bio-nano delivery systems may provide additional options for improving the precision of AD gene therapy (Liu et al., 2024). Immunomodulatory nanomaterials may also inform future approaches to immune regulation (Guo et al., 2026). Their use alongside AAV remains a future research direction and has not yet been established as a therapeutic strategy.

For AAV-based therapy, the next step is therefore not to pursue broader expression or more complex network regulation as goals in themselves. Priority should be given to quantitative dose–coverage relationships, human-relevant and nonhuman-primate validation, payload-specific safety, biomarker-defined patient selection, reproducible manufacturing and long-term clinical surveillance. Therapeutic success will ultimately require evidence that the appropriate payload reaches the appropriate cells and brain regions in the appropriate patient at a tolerable dose and produces meaningful functional benefit. This alignment, rather than delivery efficiency or durable expression alone, should define the translational readiness of AAV-based therapy for AD.
