# Supplementary material for: AAV-mediated gene therapy for Alzheimer’s disease: neuroprotective mechanisms and translational challenges
Source: Front Aging Neurosci. 2026 Sep 15;18:1901875. doi: 10.3389/fnagi.2026.1901875 (PMC13621402; doi:10.3389/fnagi.2026.1901875)
Supplement: Supplementary file 1 [file Supplementary_file_1.docx]

Supplementary Methods: Literature search, evidence identification and record handling

# 1. Review approach and information sources

This narrative review was informed by a documented literature search designed to identify therapeutic and translational evidence relevant to AAV-mediated approaches for Alzheimer disease. PubMed was searched from database inception to 24 July 2026, Web of Science Core Collection on 25 July 2026, and ClinicalTrials.gov on 25 July 2026. Citation tracking and publication-version reconciliation were completed by 11 August 2026. No date, language, species or document-type restrictions were applied to the primary publication searches. Complete database-specific search strategies and search histories are provided in Supplementary Data S2.

# 2. Search strategy

Searches combined concepts related to Alzheimer disease and adeno-associated virus with thematic terms covering therapeutic intervention, amyloid and APP biology, APOE, tau, neuroinflammation and microglia, neurotrophic and synaptic strategies, RNA interference and gene editing, delivery and biodistribution, safety, clinical development and biomarkers. Product-specific searches were also performed for CERE-110/AAV2-NGF, LX1001/AAVrh.10-APOE2 and AAV2-BDNF.

Broader literature on CNS AAV delivery, immunity, safety and manufacturing was used when directly relevant to interpretation of AD-directed approaches. ClinicalTrials.gov searches combined a broad Alzheimer disease/AAV query with programme-specific searches. Eligible registry records were linked to related publications and analysed at the programme level.

# 3. Record management and eligibility

PubMed and Web of Science records were imported into EndNote and reconciled before database merging. Duplicate and publication-version records were identified using bibliographic identifiers and manual review. The 7,511 raw database occurrences were reduced to 936 unique database records; seven additional eligible reports were identified through citation tracking and record reconciliation.

Reports were eligible if they evaluated an AAV-mediated therapeutic intervention relevant to Alzheimer disease or provided directly relevant translational evidence concerning vector performance, regulatory elements, delivery, biodistribution, human-derived systems, nonhuman-primate or large-animal validation, immunity, safety, manufacturing or payload biology. Reviews, editorials and secondary reports without unique evidence, non-AAV interventions without directly applicable translational information, superseded publication versions, and reporter or disease-modeling studies without therapeutic or translational relevance were excluded.

# 4. Evidence linkage and synthesis

Publication reports were used as screening units but were not assumed to represent independent experiments. Related reports from the same programme, cohort, experimental series, follow-up study or publication lineage were linked into Study Families. Peer-reviewed publications superseded corresponding preprints or conference abstracts when they represented the same work. Preprints and conference reports were retained when relevant but were explicitly identified as such. Conference-level clinical programme sources identified only through registry reconciliation were retained as linked programme evidence but were not added to the 363-publication-report screening count unless they had entered the publication-search workflow.

Evidence was synthesized narratively. Delivery and biodistribution, molecular target engagement, pathological effects, neuronal or synaptic outcomes, behavioral or functional outcomes, safety findings and human clinical efficacy were considered as distinct evidence domains. No formal risk-of-bias or certainty-of-evidence grading was performed.

# 5. Search and screening results

The PubMed and Web of Science searches yielded 7,511 raw record occurrences and 936 unique database records after deduplication. Seven additional reports were identified through other methods, giving 943 records for screening. Of these, 378 reports were sought for retrieval, four could not be retrieved, and 374 were assessed in full. Eleven reports were excluded after full-text assessment, leaving 363 publication reports linked into 196 publication-derived Study Families.

Six eligible ClinicalTrials.gov records representing four clinical programme families were evaluated separately. Supplementary Data S1 provides the included publication inventory, Supplementary Data S2 provides the complete search and screening audit, Supplementary Table S1 provides detailed extraction for representative therapeutic and clinical evidence, and Supplementary Figure S1 summarizes record identification and selection.
