# Supplementary material for: AAV-mediated gene therapy for Alzheimer’s disease: neuroprotective mechanisms and translational challenges
Source: Front Aging Neurosci. 2026 Sep 15;18:1901875. doi: 10.3389/fnagi.2026.1901875 (PMC13621402; doi:10.3389/fnagi.2026.1901875)
Supplement: Supplementary file 2 [file Supplementary_file_2.docx]

**Supplementary Figure S1. Diagram of literature identification, screening and evidence inclusion.** Records identified through PubMed and Web of Science Core Collection were deduplicated and screened separately from additional records identified through citation tracking and record reconciliation. Publication reports were subsequently linked into Study Families to reduce double counting of related reports, programme updates and publication versions. ClinicalTrials.gov records were evaluated separately and are not included in the publication-report counts shown here.
